# Effects of Soil Amendment Calcium Carbonate and Chiral Pesticide Tetraconazole on the Residues of Pesticide and Cadmium in Rice

**DOI:** 10.3390/toxics14090747

**Published:** 2026-08-25

**Authors:** De Chen, Ziyang Diao, Xuezhu Ye, Yuanlin Gu, Huiyu Zhao, Peipei Qi, Zhiwei Wang, Zhenzhen Liu, Xinquan Wang, Shanshan Di

**Affiliations:** 1 State Key Laboratory for Quality and Safety of Agro-Products/Key Laboratory of Detection for Pesticide Residues and Control of Zhejiang, Institute of Agro-product Safety and Nutrition, Zhejiang Academy of Agricultural Sciences, Desheng Middle Road 298, Hangzhou 310021, China; chende@zaas.ac.cn (D.C.);; 2 Agricultural Ministry Key Laboratory for Pesticide Residue Detection, Hangzhou 310021, China

**Keywords:** chiral tetraconazole, calcium carbonate, rice cultivars, environmental behaviors, soil microorganisms

## Abstract

Calcium carbonate (CaCO_3_) is a common soil amendment to alleviate cadmium (Cd) transformation and accumulation in brown rice, and tetraconazole is a widely used chiral pesticide to control rice sheath blight. This study investigated the effects of soil amendment CaCO_3_ and chiral pesticide tetraconazole on pesticide residues, Cd accumulation, and soil microorganisms in two rice cultivars (ZG 96 with low Cd accumulation and ZY 18 with high Cd accumulation) in Cd-contaminated paddy fields. Results showed that CaCO_3_ significantly reduced Cd concentrations in brown rice by 60.0–82.9%. It altered tetraconazole degradation half-lives in rice plants, increased residues in ZG 96 rice husk, and decreased enantioselectivity in soil. Tetraconazole residues in brown rice were lower in ZY 18 than in ZG 96. Both CaCO_3_ and tetraconazole reshaped soil microbial diversity, community structure, and metabolic functions, with distinct responses between the two rice cultivars. All residues of tetraconazole and Cd in brown rice were below maximum residue limits. The rice cultivar differences should be considered under the application of amendments and chiral pesticides, and this study would provide valuable data and theoretical basis for their combined treatments.

## 1. Introduction

The rapid development of industrialization and urbanization in recent years has led to the discharge of heavy metals into the environment, which has caused severe impacts on soil globally [1]. Soil heavy metal pollution in major rice-producing areas in southern China has been a matter of concern over the last several decades, with cadmium (Cd) being the major contaminant [2]. Due to the relatively high bioavailability of Cd in soil and the high capacity of rice to accumulate Cd, a large proportion of harvested rice grains exceeds safe Cd thresholds [3]. Long-term consumption of rice with excessive Cd concentration may endanger human health [4]. Therefore, it is an urgent task to remediate Cd-contaminated soil, reducing Cd content in rice grains. In situ remediation is a cost-effective way to tackle Cd-contaminated soil, which has been widely used on a large scale in China [5,6]. Liming materials, such as calcium carbonate (CaCO_3_), quick lime, and hydrated lime, have been widely used to improve soil pH and reduce the bioavailability of Cd in soil [7,8]. The application of CaCO_3_ at 2 g/kg could reduce the Cd concentration in brown rice by 69% [9].

Pesticide application has become a common practice to improve crop yield and quality [10]. Tetraconazole is a widely used sterol demethylation inhibitor with good systemic properties, which has good protective and therapeutic activities. Tetraconazole can be used to control rice sheath blight [11]. Tetraconazole has one chiral carbon atom and two chiral enantiomers. The enantiomer differences of tetraconazole have been reported, and *R*-tetraconazole showed stronger bactericidal activity than *S*-tetraconazole against two pathogens (*Rhizoctonia cerealis* and *Fusarium graminearum*) [12]. *R*-tetraconazole was preferentially degraded in wheat soil [12]. During the strawberry wine-making process and apples with bagging treatment from three regions, (-)-tetraconazole was degraded faster than (+)-tetraconazole [13,14]. The co-exposure of Cd increased the accumulation of *S*-tetraconazole, while decreasing the concentrations of *R*-tetraconazole in zebrafish [15]. However, under conditions of pesticide application in Cd-contaminated soils, whether the application of CaCO_3_ would affect the enantioselective behaviors of tetraconazole in rice needed to be studied.

This study evaluated the effects of CaCO_3_ and tetraconazole on two rice cultivars with distinct Cd accumulation abilities in a Cd-contaminated paddy field. It explored how CaCO_3_ impacts enantioselective degradation and pesticide residues, Cd accumulation in brown rice, and changes in soil microorganisms and their metabolic functions. The findings contribute to the efficient and safe application of chiral tetraconazole and CaCO_3_ and provide new insights into the effects of soil amendment on pesticides and heavy metals in the soil environment.

## 2. Materials and Methods

### 2.1. Compounds and Reagents

The racemic tetraconazole with a purity of 98% was obtained from J&K Scientific Ltd. (Shanghai, China). CaCO_3_ was purchased from Lingshou Yunda Mineral Products Co., Ltd. (Shijiazhuang, China). The other chemicals and materials are described in Supplementary Materials.

### 2.2. Field Experiment

The field experiments were carried out in a Cd-contaminated paddy field in Shaoxing, Zhejiang Province, China. Two rice cultivars with contrasting Cd accumulation abilities were selected for planting, including Zheyou 18 (ZY 18) and Zhegeng 96 (ZG 96), where the former is an indica japonica three-line hybrid rice cultivar with a relatively higher Cd accumulation capacity, and the latter is a japonica conventional rice with relatively lower Cd accumulation capacity. The experimental soil was classified as purple clayey paddy soil, and the pH, organic matter, total nitrogen, and total phosphorus were 5.0, 46.8 g/kg, 2.5 g/kg, and 0.4 g/kg, respectively. The background soil Cd concentration was 0.39 mg/kg. Eight treatments (Table 1 and Figure S1) were established, and each treatment covered an area of 100 m^2^ [16]. The treatments of KQ7d_1, KQ7d_3, KQ7d_5, and KQ7d_7 corresponded to ZG 96 exposed to Cd, Cd+CaCO_3_, Cd+tetraconazole, and Cd+CaCO_3_+tetraconazole, respectively. The treatments of KQ7d_2, KQ7d_4, KQ7d_6, and KQ7d_8 represented ZY 18 under the same four exposure conditions in sequence. Buffer zones were arranged between different treatments. CaCO_3_ was applied 10 days before rice transplanting at the dose of 4.5 t/ha, and was ploughed evenly with a depth of 0–20 cm.

In the degradation experiment, 17% tetraconazole·azoxystrobin suspension concentrate (7.5% of tetraconazole) was diluted to 48.375 g a.i./ha with water (450 L/ha) and sprayed once according to the recommended dosage. Based on the five-point method, representative rice plant samples were collected randomly at 2 h, 1, 3, 5, 7, 10, 14, 21, 28, and 35 days after application. Rice husk and brown rice were collected during the mature stage and before and after 7 days of the mature stage. All samples were separately homogenized and stored at −20 °C.

### 2.3. Sample Preparation and Analysis

For the determination of tetraconazole enantiomers, the extraction and purification of samples were adopted according to a report [17], and the Nexera UC SFC system (Shimadzu, Japan) with an LCMS-8050 (Shimadzu) was used. For Cd analysis, the plant samples were oven-dried and ground using a stainless-steel grinder. The soil samples were air-dried, ground, and passed through sieves with different mesh numbers. ICP-MS (X-series 2, Thermo, Waltham, MA, USA) was used for heavy metal analysis [6]. For soil property analysis, the soils sampled on the 7th day were used to test the soil properties according to the report [18]. All samples were treated with three repetitions. The specific pretreatment and detection methods are shown in Supplementary Materials.

### 2.4. Determination of Soil Microorganisms

According to the report, tetraconazole application significantly shifted the microbial community structure on the 7th day [19]. Thus, the soil samples on the 7th day were used to measure the soil properties and soil microorganisms. The sequencing process was based on the report, and the details were provided in Supplementary Materials [15].

### 2.5. Data Analysis

The degradation behavior of tetraconazole enantiomers is fitted by the first-order kinetics equation, and the half-life (*t*_1/2_) is calculated [15]:

$$C_{t}=C_{0}\times \exp.(-k_{d}\times t)\;\;t_{1/2}=\ln2/k_{d},$$

where *C_t_*: concentrations (ng/g) at time t, and *k_d_*: dissipation rate constant (d^−1^) of tetraconazole enantiomers (days).

Using enantiomer fraction (EF) as the indicator to measure the enantioselective degradation of tetraconazole [15]:

$$\mathrm{EF}=\frac{C_{S}}{C_{S}+C_{R}},$$

where *C_S_*: concentrations of *S*-(+)-tetraconazole (ng/g), and *C_R_*: concentrations of *R*-(-)-tetraconazole (ng/g). EF = 0.5 indicates that there is no enantioselectivity, EF < 0.5 indicates that *S*-(+)-tetraconazole is degraded preferentially, and EF > 0.5 indicates that *R*-(-)-tetraconazole is degraded preferentially.

Differential analysis of tetraconazole concentrations, Cd concentrations, and soil properties were carried out using SPSS software (version 20.0; one-way ANOVA and Tukey, *p* < 0.05).

## 3. Results

### 3.1. Enantioselective Fate of Tetraconazole Enantiomers in Rice Planting System

The degradation of tetraconazole enantiomers in rice plants under all treatment conditions was fit to the first-order degradation kinetics, and the parameters and half-lives are listed in Table 2.

#### 3.1.1. ZG 96 Rice Plants

**Figure 1:**
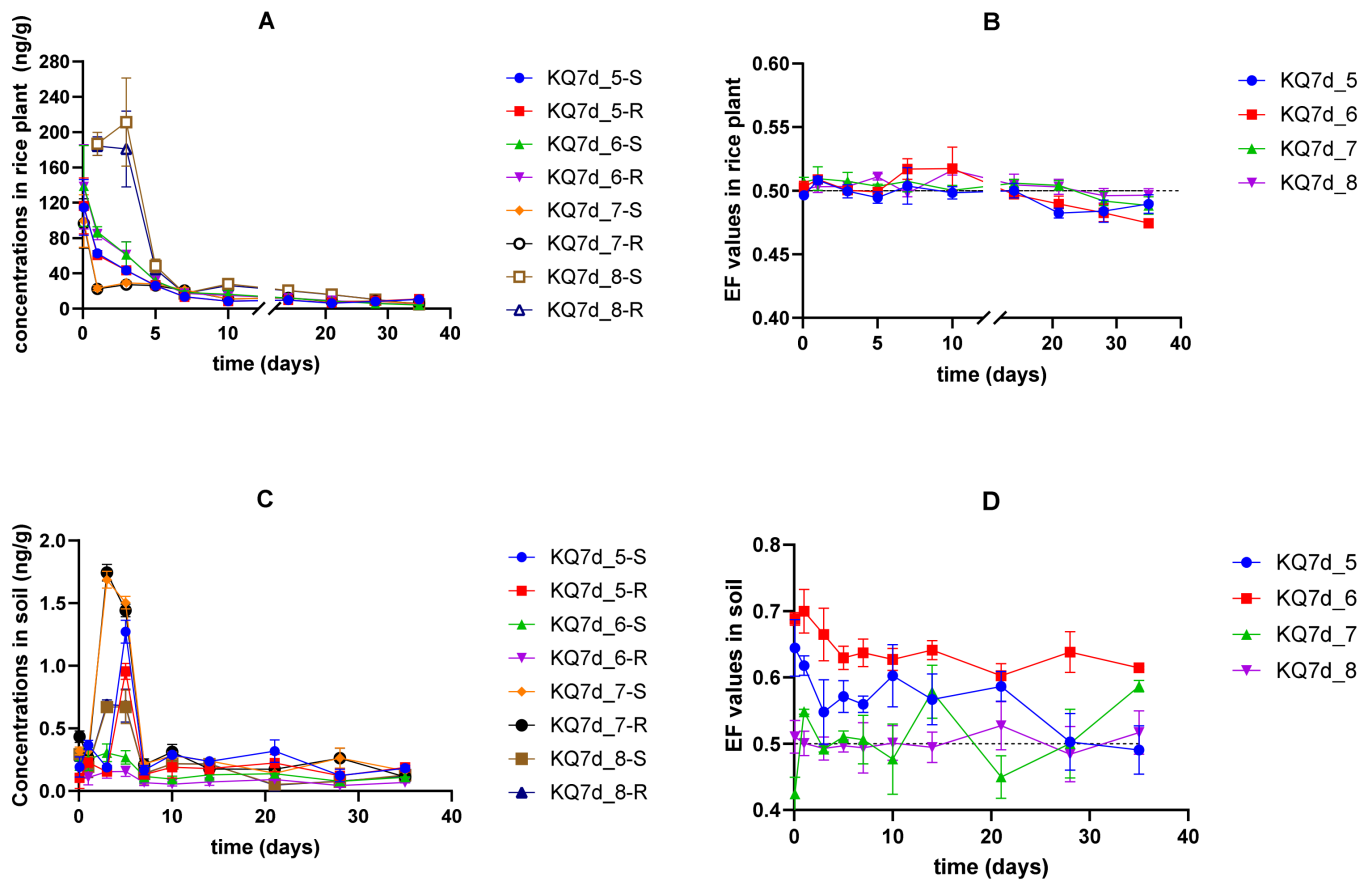
The concentration curves and EF values of tetraconazole enantiomers in rice plants (**A**,**B**) and soil (**C**,**D**) (means ± SD, n = 3). The treatments of KQ7d_5 and KQ7d_7 were the exposure of Cd+tetraconazole and Cd+CaCO_3_+tetraconazole on ZG 96, respectively. The treatments of KQ7d_6 and KQ7d_8 were the exposure of Cd+tetraconazole and Cd+CaCO_3_+tetraconazole on ZY 18, respectively. R and S represent the concentrations of R-tetraconazole and S-tetraconazole, respectively.

The initial concentration of *S*-(+)- and *R*-(-)-tetraconazole in ZG 96 rice plants (KQ7d_5) was 115 and 116 ng/g, respectively (Figure 1A). In the plot with CaCO_3_ (KQ7d_7), the initial concentration of *S*-(+)- and *R*-(-)-tetraconazole was 99.3 and 96.4 ng/g, respectively, but the half-lives were changed from 2.14–2.18 days to 2.23–2.41 days. No matter whether CaCO_3_ was added or not, tetraconazole enantiomers did not undergo enantioselective degradation in rice plants (Figure 1B).

#### 3.1.2. ZY 18 Rice Plants

The initial concentrations of *S*-(+)- and *R*-(-)-tetraconazole in ZY 18 rice plants (KQ7d_6) were 139 and 138 ng/g, respectively (Figure 1A). In the plot with CaCO_3_ (KQ7d_8), the initial concentration of *S*-(+)- and *R*-(-)-tetraconazole increased, which was 187 and 184 ng/g, respectively. The half-lives changed from 2.47–2.49 days to 1.86–1.98 days, which were shortened by 16.6–17.8%. No enantioselective degradation was found (Figure 1B).

#### 3.1.3. Paddy Soil

During 3–5 days, the tetraconazole enantiomer concentrations in paddy soil with CaCO_3_ (KQ7d_7 and KQ7d_8, 0.67–1.74 ng/g) were higher than those in soil without CaCO_3_ (KQ7d_5 and KQ7d_6, 0.19–1.27 ng/g; Figure 1C). The concentration of *R*-(-)-tetraconazole was always lower than *S*-(+)-tetraconazole in the paddy soil without CaCO_3_ (KQ7d_5 and KQ7d_6) with EF values > 0.5 (Figure 1D). However, the concentrations of *S*-(+)- and *R*-(-)-tetraconazole in the soil with CaCO_3_ (KQ7d_7 and KQ7d_8) had little difference. CaCO_3_ decreased the enantioselectivity of tetraconazole in paddy soil.

### 3.2. Enantioselective Residue of Tetraconazole in Rice Husk and Brown Rice

**Figure 2:**
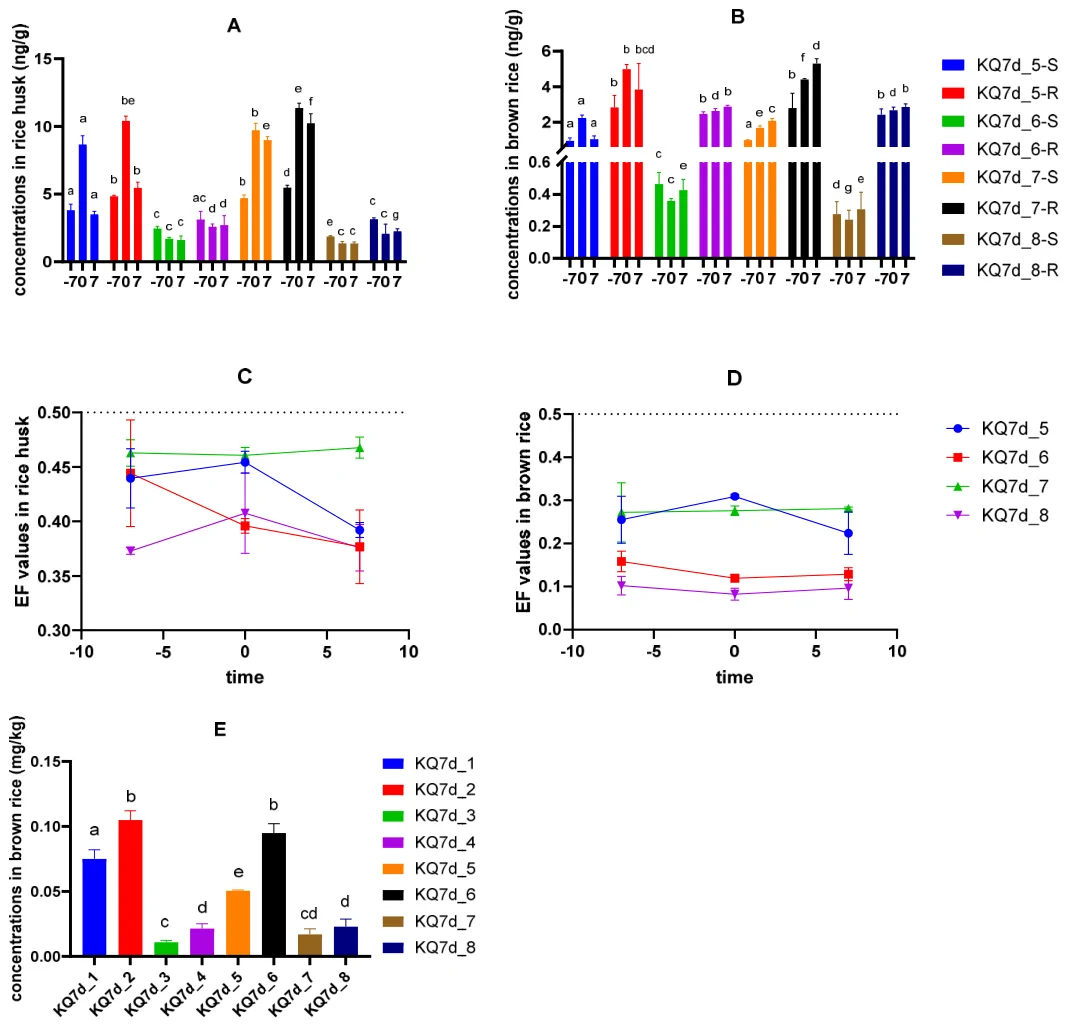
The residue concentrations and EF values of tetraconazole enantiomers in rice husk (**A**,**C**) and brown rice (**B**,**D**) (means ± SD, n = 3). The residue concentrations of Cd in brown rice (**E**) (means ± SD, n = 3). The different letters indicate significant differences among different treatments on the same day (*p* < 0.05). The treatments of KQ7d_1, KQ7d_3, KQ7d_5, and KQ7d_7 were the exposure of Cd, Cd+CaCO_3_, Cd+tetraconazole, and Cd+CaCO_3_+tetraconazole on ZG 96, respectively. The treatments of KQ7d_2, KQ7d_4, KQ7d_6, and KQ7d_8 were the exposure of Cd, Cd+CaCO_3_, Cd+tetraconazole, and Cd+CaCO_3_+tetraconazole on ZY 18, respectively. R and S represent the concentrations of R-tetraconazole and S-tetraconazole, respectively, and 0, −7, and 7 represent the mature stage and before and after 7 days of the mature stage, respectively.

CaCO_3_ enhanced the residual tetraconazole concentration in ZG 96 rice husk by 10.5–115% but decreased the tetraconazole residue by 10.0–19.2% in ZY 18 rice husk (Figure 2). The concentrations of *S*-(+)-tetraconazole were always less than *R*-(-)-tetraconazole in all treatments, and the EF values were all <0.5. *R*-(-)-tetraconazole was more easily accumulated in rice husk and brown rice. The addition of CaCO_3_ did not cause a significant difference in the enantioselectivity of tetraconazole. For different rice cultivars, the residues of tetraconazole in ZG 96 brown rice were 1.29–2.41 times those of ZY 18, and in ZG 96 rice husk they were 1.55–6.12 times those of ZY 18. The maximum residue limit (MRL) of tetraconazole in rice stipulated by the European Food Safety Authority was 0.01 mg/kg. The residual tetraconazole concentrations in brown rice in all treatments were less than the MRL.

### 3.3. Cd Concentration in Brown Rice

In the plots with CaCO_3_ (Figure 2E), the Cd contents in brown rice decreased significantly. Compared with the Cd-alone treatments (KQ7d_1 and KQ7d_2), the concentrations of Cd in the treatments with CaCO_3_ (KQ7d_3 and KQ7d_4) in brown rice were reduced by 82.9% and 78.2%, respectively. Compared with the treatments of Cd and tetraconazole (KQ7d_5 and KQ7d_6), the treatments with CaCO_3_ (KQ7d_7 and KQ7d_8) decreased the Cd concentrations in brown rice by 60.0–81.0%. The concentration of Cd in ZY 18 was 1.35–1.95 times that of ZG 96 in different treatments, showing a stronger ability to accumulate Cd in ZY18. The MRLs of Cd in brown rice stipulated by China and the EU are both 0.2 mg/kg [20]. Fortunately, the residual amounts of Cd in brown rice in all treatments were less than the MRL.

### 3.4. Changes in Soil Properties

After using CaCO_3_, the soil pH increased from 5.1–5.5 to 6.0–6.4 (Figure S2A). The organic matter contents had little change (Figure S2B). The contents of total nitrogen and available nitrogen increased by 0.09–0.3 g/kg and 8.35–47.8 mg/kg, respectively, after applying tetraconazole (Figure S2C,D). The alone application of CaCO_3_ or tetraconazole had little effect on the total phosphorus and available phosphorus, which were significantly increased (0.045–0.050 g/kg and 2.2–3.4 mg/kg, respectively) after the combined application of CaCO_3_ and tetraconazole (Figure S2E,F).

### 3.5. Changes in Soil Microorganisms

#### 3.5.1. Alpha and Beta Diversity Analysis

**Figure 3:**
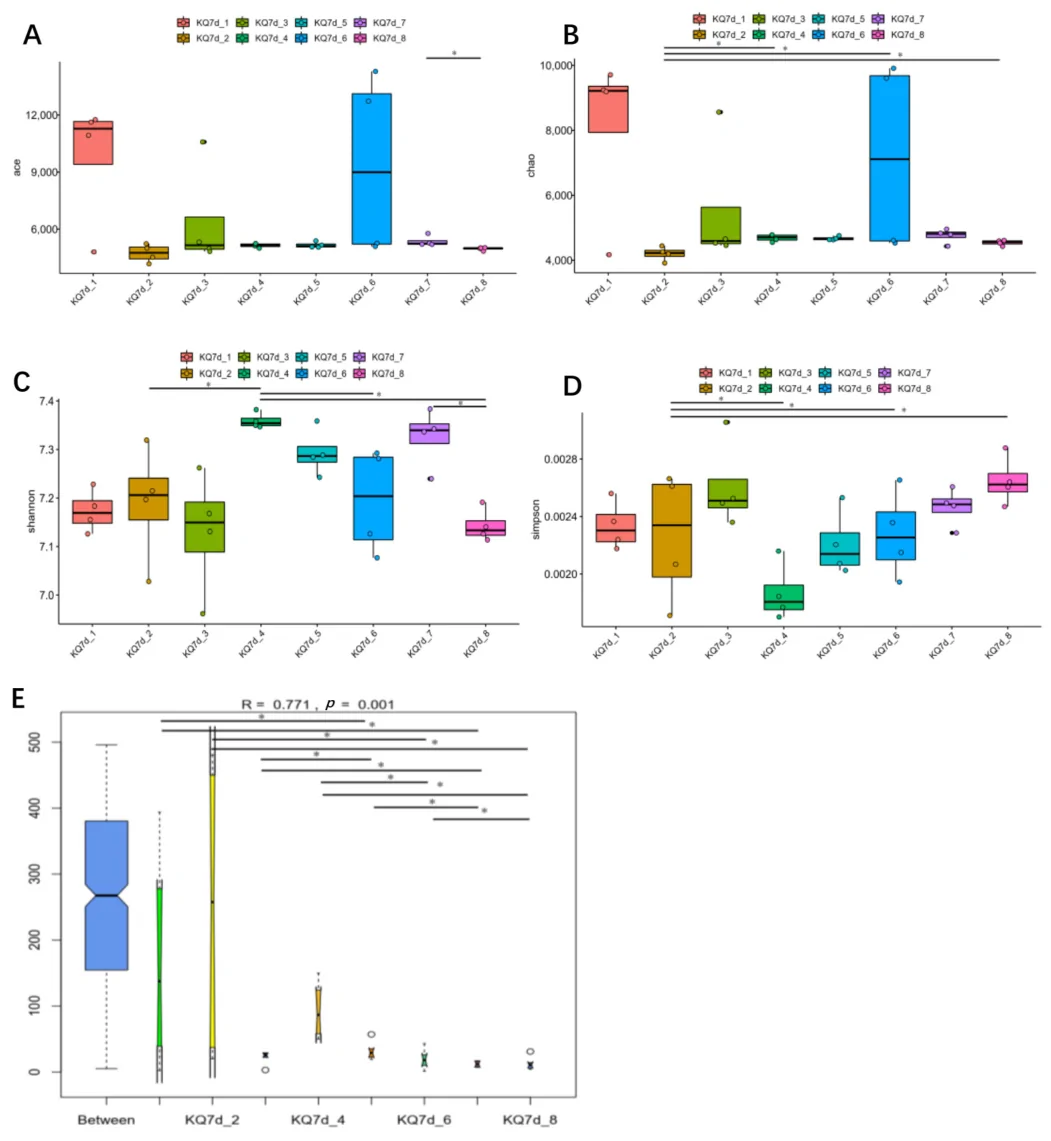
Alpha diversity analysis of soil microorganisms in different treatments, including Ace (**A**), Chao (**B**), Shannon (**C**), and Simpson indices (**D**). The ANOSIM analysis was used to analyze the degree of dissimilarity and diversity among soil microorganisms in different treatments (**E**). * Indicates a significant difference (*p* < 0.05). The treatments of KQ7d_1, KQ7d_3, KQ7d_5, and KQ7d_7 were the exposure of Cd, Cd+CaCO_3_, Cd+tetraconazole, and Cd+CaCO_3_+tetraconazole on ZG 96, respectively. The treatments of KQ7d_2, KQ7d_4, KQ7d_6, and KQ7d_8 were the exposure of Cd, Cd+CaCO_3_, Cd+tetraconazole, and Cd+CaCO_3_+tetraconazole on ZY 18, respectively.

For Ace (Figure 3A) and Chao indices (Figure 3B), there was a decrease in KQ7d_3, KQ7d_5, and KQ7d_7 treatments compared with KQ7d_1, and a significant increase in KQ7d_4, KQ7d_6, and KQ7d_8 compared with KQ7d_2. The Shannon indices (Figure 3C) in KQ7d_4 compared with KQ7d_2, and KQ7d_6 and KQ7d_8 compared with KQ7d_4, increased significantly, indicating an increase in microbial diversity. For Simpson indices (Figure 3D), there was a significant decrease in KQ7d_4, KQ7d_6, and KQ7d_8 compared with KQ7d_2. Beta diversity analysis (Figure S3) indicated that the samples were similar when the distribution between different treatments was close. The principal coordinate analysis (PCoA) indicated that PCoA1 = 27.68% and PCoA2 = 20.10% (Figure S3). Moreover, the KQ7d_2 treatment was completely separated from KQ7d_7 and KQ7d_8, and the KQ7d_7 treatment was completely separated from KQ7d_3 and KQ7d_8.

#### 3.5.2. Inter-Group Difference Analysis

ANOSIM analysis was used to test whether the differences between groups were significantly greater than those within groups. The ANOSIM analysis based on the binary Bray–Curtis method showed a significant difference (R = 0.771, *p* = 0.001) in microbial diversity among different groups except for KQ7d_1 vs. KQ7d_2 and KQ7d_2 vs. KQ7d_4 (Figure 3E), implying that different treatments might induce varying impact degrees on soil microorganisms.

#### 3.5.3. Relative Abundance Changes of Microorganisms

##### Phylum Level

**Figure 4:**
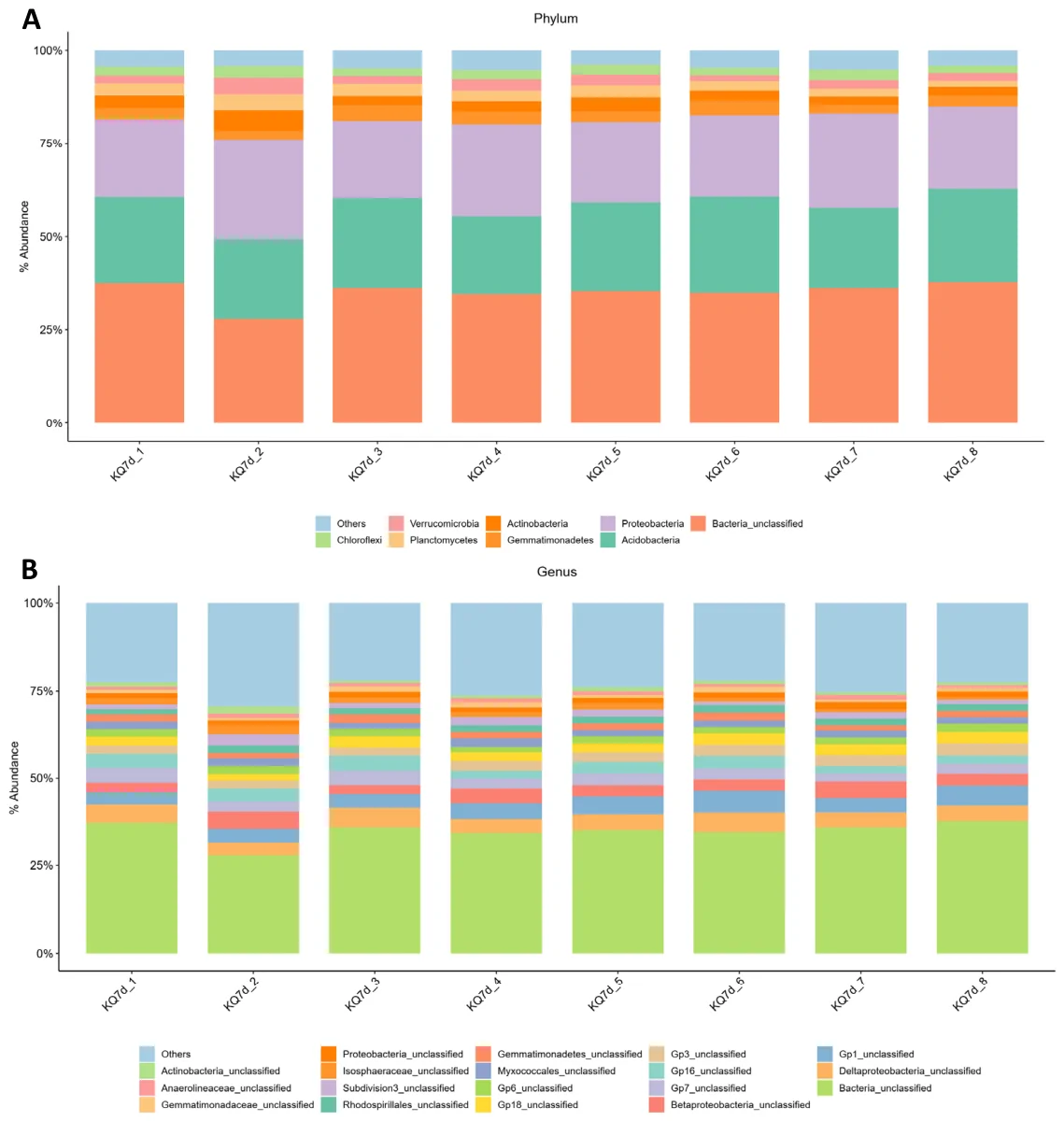
The community composition and abundance analysis at the phylum (**A**) and genus (**B**) levels. The treatments of KQ7d_1, KQ7d_3, KQ7d_5, and KQ7d_7 were the exposure of Cd, Cd+CaCO_3_, Cd+tetraconazole, and Cd+CaCO_3_+tetraconazole on ZG 96, respectively. The treatments of KQ7d_2, KQ7d_4, KQ7d_6, and KQ7d_8 were the exposure of Cd, Cd+CaCO_3_, Cd+tetraconazole, and Cd+CaCO_3_+tetraconazole on ZY 18, respectively.

At the phylum level, the top eight microorganisms were *Bacteria_unclassified*, *Acidobacteria*, *Proteobacteria*, *Planctomycetes*, *Actinobacteria*, *Gemmatimonadetes*, *Chloroflexi*, and *Verrucomicrobia* (Figure 4A and Table S1). For ZG 96, compared with KQ7d_1 treatment, CaCO_3_ (KQ7d_3) treatment enhanced the relative abundance of *Acidobacteria* and *Gemmatimonadetes*, and reduced the relative abundance of *Proteobacteria* and *Chloroflexi*, while the opposite effects were observed in CaCO_3_+tetraconazole (KQ7d_7) combined treatment. The increase in relative abundance of Actinobacteria was observed in tetraconazole (KQ7d_5) treatment, while a decrease was observed in KQ7d_3 and KQ7d_7 treatments. KQ7d_5 and KQ7d_7 treatments increased the relative abundance of *Verrucomicrobia*.

For ZY 18, compared with KQ7d_2 treatment, the increased relative abundance of *Bacteria_unclassified* and *Gemmatimonadetes*, and the decrease in relative abundance of *Proteobacteria*, *Planctomycetes*, *Actinobacteria*, *Chloroflexi*, and *Verrucomicrobia*, were observed in all treatments (KQ7d_4, KQ7d_6, and KQ7d_8). The decrease in relative abundance of *Acidobacteria* was observed in the KQ7d_4 (CaCO_3_) treatment, while the increase was observed in the KQ7d_6 and KQ7d_8 (tetraconazole and CaCO_3_+tetraconazole) treatments.

##### Genus Level

As illustrated in Figure 4B and Table S2, for ZG 96, compared with the KQ7d_1 treatment, the CaCO_3_+tetraconazole (KQ7d_7) treatment reduced the relative abundance of *Gp7_unclassified* in KQ7d_3 and KQ7d_5 treatments, exhibiting a synergistic inhibition effect, and enhanced the relative abundance of *Proteobacteria_unclassified*, exhibiting a synergistic promotion effect. The effects of CaCO_3_ (KQ7d_3) and tetraconazole (KQ7d_5) were opposite on the relative abundance of *Deltaproteobacteria_unclassified*, *Gp16_unclassified*, *Gemmatimonadetes*, and *Actinobacteria_unclassified*, and the combined treatments (KQ7d_7) showed inhibitory effects. The decrease in relative abundance of *Bacteria_unclassified*, *Isosphaeraceae_unclassified*, and *Chloroflexi_unclassified*, and the increase in relative abundance of *Rhodospirillales_unclassified* and *Proteobacteria_unclassified*, were observed in all treatments (KQ7d_3, KQ7d_5, and KQ7d_7).

For ZY 18, compared with the KQ7d_2 treatment, the KQ7d_8 (CaCO_3_+tetraconazole) treatment decreased the relative abundance of *Isosphaeraceae_unclassified* in KQ7d_4 (CaCO_3_) and KQ7d_6 (tetraconazole) treatments, exhibiting a synergistic inhibition effect, and enhanced the relative abundance of *Bacteria_unclassified*, *Gp3_unclassified*, and *Proteobacteria_unclassified*, exhibiting a synergistic promotion effect. The decrease in relative abundance of *Gp6_unclassified* in the KQ7d_8 treatment was weaker than that in KQ7d_4 and KQ7d_6 treatments. For *Actinobacteria_unclassified*, *Rhizobiales_unclassified*, and *Chloroflexi_unclassified*, the decrease in relative abundance in the KQ7d_8 treatment was similar to that in KQ7d_4 and KQ7d_6 treatments. The decrease in *Gp16_unclassified* relative abundance in the KQ7d_8 treatment might be mainly caused by CaCO_3_. The increase in relative abundance of *Gp1_unclassified*, *Deltaproteobacteria_unclassified*, *Gemmatimonadetes_unclassified*, and *Gp18_unclassified*, and the decrease in relative abundance of *Betaproteobacteria_unclassified* and *Subdivision3_unclassified*, in KQ7d_8 treatment might be mainly caused by tetraconazole.

#### 3.5.4. KEGG Function Prediction

**Figure 5:**
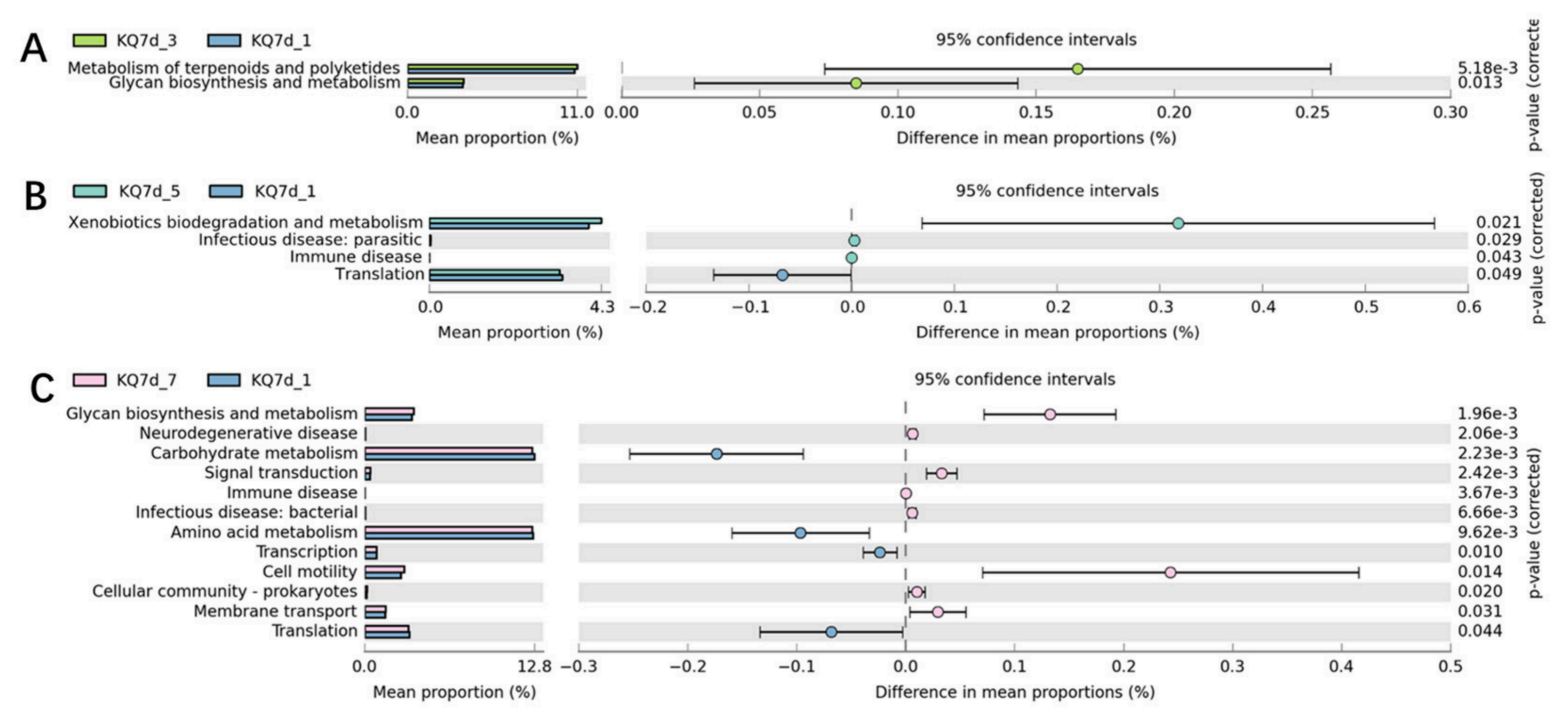
The prediction analysis of KEGG pathways between different treatment groups at KEGG level 2: KQ7d_1 vs. KQ7d_3 (**A**), KQ7d_1 vs. KQ7d_5 (**B**), and KQ7d_1 vs. KQ7d_7 (**C**). The treatments of KQ7d_1, KQ7d_3, KQ7d_5, and KQ7d_7 were the exposure of Cd, Cd+CaCO_3_, Cd+tetraconazole, and Cd+CaCO_3_+tetraconazole on ZG 96, respectively.

For ZG 96, the addition of CaCO_3_ affected metabolism of terpenoids and polyketides and glycan biosynthesis and metabolism (Figure 5A). Tetraconazole-alone treatment affected xenobiotics biodegradation and metabolism (Figure 5B). CaCO_3_+tetraconazole combined treatment affected glycan biosynthesis and metabolism, signal transduction, cell motility, and membrane transport (Figure 5C). Compared to CaCO_3_-alone treatment, tetraconazole-alone treatment affected glycan biosynthesis and metabolism, nucleotide metabolism, and folding, sorting, and degradation (Figure S4C). Compared to tetraconazole-alone treatment, CaCO_3_+tetraconazole combined treatment affected cell growth and death, cellular community prokaryotes, and glycan biosynthesis and metabolism (Figure S4D).

For ZY 18, tetraconazole treatment affected cellular community prokaryotes and glycan biosynthesis and metabolism (Figure S4A). CaCO_3_+tetraconazole combined treatment affected cellular community prokaryotes, cell growth and death, energy metabolism, metabolism of cofactors and vitamins, and glycan biosynthesis and metabolism (Figure S4B).

## 4. Discussion

Cd pollution risk in rice planting systems has always been a hot spot of continuous concern. Using amendments (lime, biochar, clay mineral, organic fertilizer, etc.) and planting rice cultivars with low accumulation ability of heavy metals (DMY6188, DX4103, NY6368, GY725, etc.) are normally used to reduce the uptake and transfer of Cd from soil to rice, which can reduce the accumulation of Cd by more than 70% [21,22,23]. However, it is inevitable to use pesticides during rice planting. Whether the use of soil amendments affects the residue and degradation of pesticides and whether the mixed use of them affects soil microorganisms is worthy of attention.

According to the report, the half-life of tetraconazole in rice plants was 2.1–6.3 days [24]. In this study, the half-lives of tetraconazole in rice plants (KQ7d_5 and KQ7d_6 treatments) were 2.14–2.49 days, which was similar to the report. The half-lives of tetraconazole were 10.3–13.6 days in wheat soil [12]. In this study, tetraconazole was also degraded rapidly in seven days. However, considering the test soil was Cd-contaminated soil and there was no without-Cd soil in the test area, the Cd effects on the tetraconazole degradation were not tested simultaneously. Studies have shown that the presence of heavy metals will affect the environmental behavior of pesticides. For example, the co-existence of Cd^2+^ could prolong the persistence of atrazine in water [25]. Cd^2+^ and Pb^2+^ would inhibit the degradation of α-cypermethrin in soil, which might be related to the activity of soil microbes or the level of enzymes [26].

The soil amendments may also affect the pesticide fate. Liu et al. reported that the application of urea and phosphate fertilizers would promote the degradation of penthiopyrad in soil [27]. Here, the addition of CaCO_3_ increased the maximum concentration of tetraconazole in both ZY 18 soil and ZG 96 soil and decreased the enantioselectivity. In rice husk, CaCO_3_ increased the tetraconazole residue for ZG 96 (10.5–115%). For cultivar differences, the residual concentrations of tetraconazole in ZY 18 plants, rice husk, and brown rice were lower than in ZG 96. In paddy soil, rice husk, and brown rice, the enantioselectivity of tetraconazole in ZY 18 was more obvious than in ZG 96. Chang et al. found that R-tebuconazole was preferentially degraded in Fuji and Hanfu apples, while no enantioselectivity was found in Huahong variety apples [28]. Similar phenomena were found in different cucumbers for mefentrifluconazole [29,30]. These differences might be caused by the different enzyme activities and contents, and the different translocation [31,32,33]. Therefore, it is necessary to study chiral pesticides at the enantiomeric level, especially for different cultivars.

The effectiveness of CaCO_3_ in reducing Cd concentrations in brown rice has been verified, and the effects of other substances have been studied [34,35]. The combined application of EDTA-Fe(II) and CaCO_3_ could effectively decrease Cd accumulation in brown rice [9]. The combined application of humic substances and CaCO_3_ had a better effect than single treatments [36]. However, the information on the pesticide effect is limited. Akter et al. found that pesticide application increased the heavy metal concentrations in soil, which indirectly and directly affected the soil properties [37]. Here, the tetraconazole application increased Cd concentration in brown rice after CaCO_3_ treatments, and significantly increased total nitrogen, available nitrogen, total phosphorus, and available phosphorus by 8.0–82.0% in soils. Kuang et al. found that increasing pH and phosphorus in soil were two important factors decreasing available Cd [38]. The application of phosphorus fertilizer significantly decreased the Cd concentrations in brown rice by about 83.0%, and the phosphorus in soil might prevent the transport of Cd to aboveground mainly by inhibiting absorption and increasing root fixation [39]. Similarly, reasonable nitrogen application is a promising strategy for reducing crop Cd accumulation, and the application of NH_4_NO_3_ and NH_4_Cl inhibited the instantaneous root Cd uptake [40]. The combined application of CaCO_3_ and tetraconazole helped improve soil fertility, which also affected the soil microorganisms.

Microorganisms play a versatile ecophysiological role in soil formation, resource cycling, and management, such as enhancing the degradation of organic pollutants in soils, promoting plant growth, and increasing soil fertility [41,42,43,44]. Microorganisms can also increase phytoremediation efficiency by solubilizing heavy metals and promoting plant growth through the production of phytohormones in heavy-metal-contaminated soils [45]. Here, the dominant microflora in soil were *Acidobacteria* and *Proteobacteria*. *Acidobacteria* is one of the most abundant phyla in soil, and has been confirmed to actively interact with plants, functioning as plant-growth-promoting bacteria [46]. *Proteobacteria*, a prevalent phylum in metal-contaminated soils, can promote the bioreduction of metals in contaminated systems by manipulating microbial diversity, having a high detoxifying effect on heavy metals [47,48]. Some sequences of *Proteobacteria* can assist in carbonate mineral formation, offering some resistance to heavy metals [38,49].

Research has shown that pesticides can change the composition of rhizosphere microorganisms [50,51]. The response of soil microorganisms to tetraconazole depended on the soil management and soil properties [19,52]. The relative abundance of *Actinobacteria*, *Chloroflexi*, and *Verrucomicrobia* increased after the application of tetraconazole for ZG 96, which was the opposite for ZY 18. *Actinobacteria* can produce secondary metabolites, which play a positive role in bioremediation and promoting plant growth efficiency [53]. *Chloroflexi* seems to play a significant beneficial role by providing filamentous scaffolding, fermenting carbohydrates, and degrading complex polymeric organic compounds into low-molecular-weight substrates to support their growth and that of other bacterial populations [54]. *Verrucomicrobia* have potential roles in methane oxidation, polysaccharide degradation, and nitrogen fixation [55,56]. These changes might affect xenobiotics biodegradation and metabolism and glycan biosynthesis and metabolism for ZG 96 soils, and affect cellular community prokaryotes and glycan biosynthesis and metabolism for ZY 18 soils, leading to different concentration changes of Cd and tetraconazole.

Soil amendment application affects soil microorganisms, as has been proven. Lime application increased the abundance and richness of the prokaryotic microbes in paddy soil, which was caused by the changes in soil properties [57]. CaCO_3_ application for ZG 96 decreased the relative abundance of *Actinobacteria* and *Proteobacteria*, while increasing the relative abundance of *Gemmatimonadetes*, stimulating the function of metabolism of terpenoids and polyketides and glycan biosynthesis and metabolism. Similar effects of CaCO_3_ were observed for ZY 18 soil microorganisms. Most terpenoids have antibacterial and bactericidal effects, and some chemical defense substances can effectively protect themselves from pathogens [58]. The *Proteobacteri*a communities are associated with functions such as ureolysis, nitrogen fixation, nitrate reduction, and hydrocarbon degradation [59]. Tian et al. speculated that Fe-Mn-modified biochar might increase soil pH by affecting the relative abundance of *Acidobacteria*, Gp1, and Gp3, and then reduce the content of available Cd in soil [60]. Thus, we speculated that the increase in soil pH and the decrease in Cd accumulation might be linked to the relative abundance changes of *Acidobacteria*, *Proteobacteria*, *Gp1*, and *Gp3*.

The combined application of CaCO_3_ and tetraconazole decreased the relative abundance of *Planctomycetes* and *Actinobacteria* for both ZG 96 and ZY 18 soils, which might be related to changes in the functions of cell growth and death, cellular community prokaryotes, and glycan biosynthesis and metabolism. *Planctomycetes* can utilize nitrite or oxidize ammonium ions to generate nitrogen, promoting global development [61]. *Actinobacteria* play an important role in nitrogen and phosphorus cycling [62], which might be related to the increase of total nitrogen, available nitrogen, total phosphorus, and available phosphorus. However, the effects on *Acidobacteria*, *Proteobacteria*, *Gemmatimonadetes*, *Chloroflexi*, and *Verrucomicrobia* were opposite for ZG 96 and ZY 18 soils. The functional changes in energy metabolism and metabolism of cofactors and vitamins were only observed for ZY 18 soils. In the same field and test conditions, rice cultivar differences were the main reason for microorganism differences. Crop cultivars differ in their root exudate profiles, architecture, and tissue chemistry, and these differences may lead to distinct microbiomes, which may explain 15% of the variation in soil microorganisms [63]. The different root microorganism compositions at the phylum level between the two rice cultivars have been reported [64]. The differences in root exudates induce plants to recruit different partners, resulting in variations in soil microorganism compositions, which deserve further study.

## 5. Conclusions

The combined effects of CaCO_3_ and tetraconazole were generally opposite for ZG 96 and ZY 18, with more significant effects for ZG 96. The combined treatment of CaCO_3_ and tetraconazole shortened the tetraconazole half-lives in ZY18 rice plants by 16.6–17.8%, which was opposite in ZG 96 rice plants. CaCO_3_ decreased the tetraconazole residue by 10.0–19.2% in ZY 18 rice husk, but not in ZG 96 rice husk. The Cd concentrations in ZY 18 brown rice were 1.35–1.95 times higher than in ZG 96. Tetraconazole and CaCO_3_ had opposite effects on *Verrucomicrobia* in ZG 96 soil and *Acidobacteria* in ZY 18 soil. The changes in the relative abundance of soil microorganisms might change the soil properties and the degradation and residues of Cd/tetraconazole. The findings contribute to the efficient and safe application of chiral tetraconazole and provide valuable data and a theoretical basis for the effect of amendments on pesticides and heavy metals in the environment. The limitation was that the field experiments were conducted at only one location during a single growing season, without replication across years or geographical locations. The amendment effects on the behaviors of pesticides and heavy metals deserve further study.

## Figures and Tables

**Table 1:** The experimental design for CaCO_3_ and tetraconazole in Cd-contaminated soil.

Treatment	Rice Variety	Cultivation Project
KQ7d_1	ZG 96	Cd
KQ7d_2	ZY 18	Cd
KQ7d_3	ZG 96	Cd+CaCO_3_
KQ7d_4	ZY 18	Cd+CaCO_3_
KQ7d_5	ZG 96	Cd+Tetraconazole
KQ7d_6	ZY 18	Cd+Tetraconazole
KQ7d_7	ZG 96	Cd+CaCO_3_+Tetraconazole
KQ7d_8	ZY 18	Cd+CaCO_3_+Tetraconazole

**Table 2:** The degradation kinetic parameters and half-lives of tetraconazole enantiomers in rice plants. The treatments of KQ7d_5 and KQ7d_7 were the exposure of Cd+tetraconazole and Cd+CaCO_3_+tetraconazole on ZG 96, respectively. The treatments of KQ7d_6 and KQ7d_8 were the exposure of Cd+tetraconazole and Cd+CaCO_3_+tetraconazole on ZY 18, respectively.

Treatment	Enantiomer	*C*_(t)_ = *C*_0_ × exp(−*k_d_* × *t*)					
		*C*_0_ (ng/g)	Standard Error	*k_d_* (day^−1^)	Standard Error	R^2^	*t*_1/2_ (Day)
KQ7d_5	*S*-(+)-tetraconazole	108	8	0.319	0.050	0.9326	2.18
	*R*-(-)-tetraconazole	108	9	0.324	0.060	0.9199	2.14
KQ7d_6	*S*-(+)-tetraconazole	133	7	0.279	0.033	0.9645	2.49
	*R*-(-)-tetraconazole	131	8	0.281	0.036	0.9590	2.47
KQ7d_7	*S*-(+)-tetraconazole	77.2	15.4	0.288	0.121	0.6162	2.41
	*R*-(-)-tetraconazole	76.1	15.3	0.311	0.133	0.6087	2.23
KQ7d_8	*S*-(+)-tetraconazole	221	27	0.351	0.092	0.8711	1.98
	*R*-(-)-tetraconazole	208	20	0.373	0.079	0.9138	1.86

## Data Availability

The original contributions presented in this study are included in the article/Supplementary Materials. Further inquiries can be directed to the corresponding authors.

## Supplementary Materials

The following supporting information can be downloaded at: https://www.mdpi.com/article/10.3390/toxics14090747/s1. The details of materials and methods. Figure S1: The experimental design schematic diagram. The treatments of KQ7d_1, KQ7d_3, KQ7d_5, and KQ7d_7 were the exposure of Cd, Cd+CaCO_3_, Cd+tetraconazole, and Cd+CaCO_3_+tetraconazole on ZG 96, respectively. The treatments of KQ7d_2, KQ7d_4, KQ7d_6, and KQ7d_8 were the exposure of Cd, Cd+CaCO_3_, Cd+tetraconazole, and Cd+CaCO_3_+tetraconazole on ZY 18, respectively. Figure S2: The soil properties in different treatments. The different letters indicate the significant differences among different treatments (*p* < 0.05). The treatments of KQ7d_1, KQ7d_3, KQ7d_5, and KQ7d_7 were the exposure of Cd, Cd+CaCO_3_, Cd+tetraconazole, and Cd+CaCO_3_+tetraconazole on ZG 96, respectively. The treatments of KQ7d_2, KQ7d_4, KQ7d_6, and KQ7d_8 were the exposure of Cd, Cd+CaCO_3_, Cd+tetraconazole, and Cd+CaCO_3_+tetraconazole on ZY 18, respectively. Figure S3: Principal coordinate analysis (PCoA) of weighted UniFrac scores of soil microorganisms in different treatments. The treatments of KQ7d_1, KQ7d_3, KQ7d_5, and KQ7d_7 were the exposure of Cd, Cd+CaCO_3_, Cd+tetraconazole, and Cd+CaCO_3_+tetraconazole on ZG 96, respectively. The treatments of KQ7d_2, KQ7d_4, KQ7d_6, and KQ7d_8 were the exposure of Cd, Cd+CaCO_3_, Cd+tetraconazole, and Cd+CaCO_3_+tetraconazole on ZY 18, respectively. Figure S4: The prediction analysis of KEGG pathways between different treatment groups at KEGG level 2: KQ7d_2 vs. KQ7d_6 (A), KQ7d_2 vs. KQ7d_8 (B), KQ7d_3 vs. KQ7d_5 (C), and KQ7d_5 vs. KQ7d_7 (D). The treatments of KQ7d_3, KQ7d_5, and KQ7d_7 were the exposure of Cd+CaCO_3_, Cd+tetraconazole, and Cd+CaCO_3_+tetraconazole on ZG 96, respectively. The treatments of KQ7d_2, KQ7d_6, and KQ7d_8 were the exposure of Cd, Cd+tetraconazole, and Cd+CaCO_3_+tetraconazole on ZY 18, respectively. Table S1: The relative abundance changes of microorganisms at the phylum level in different treatments. The treatments of KQ7d_1, KQ7d_3, KQ7d_5, and KQ7d_7 were the exposure of Cd, Cd+CaCO_3_, Cd+tetraconazole, and Cd+CaCO_3_+tetraconazole on ZG 96, respectively. The treatments of KQ7d_2, KQ7d_4, KQ7d_6, and KQ7d_8 were the exposure of Cd, Cd+CaCO_3_, Cd+tetraconazole, and Cd+CaCO_3_+tetraconazole on ZY 18, respectively. Table S2: The relative abundance changes of microorganisms at the genus level in different treatments. The treatments of KQ7d_1, KQ7d_3, KQ7d_5, and KQ7d_7 were the exposure of Cd, Cd+CaCO_3_, Cd+tetraconazole, and Cd+CaCO_3_+tetraconazole on ZG 96, respectively. The treatments of KQ7d_2, KQ7d_4, KQ7d_6, and KQ7d_8 were the exposure of Cd, Cd+CaCO_3_, Cd+tetraconazole, and Cd+CaCO_3_+tetraconazole on ZY 18, respectively.
