# Supplementary material for: Effects of Soil Amendment Calcium Carbonate and Chiral Pesticide Tetraconazole on the Residues of Pesticide and Cadmium in Rice
Source: Toxics. 2026 Aug 25;14(9):747. doi: 10.3390/toxics14090747 (PMC13610996; doi:10.3390/toxics14090747)
Supplement: Supplementary file 1 [file toxics-14-00747-s001.zip › toxics-4476810-supplementary.pdf]

Supplementary Material

**Effects of Soil Amendment Calcium Carbonate and Chiral Pesticide Tetraconazole on the Residues of Pesticide and Cadmium in Rice**

De Chen [1,2,†], Ziyang Diao [1,2,†], Xuezhu Ye [1,2], Yuanlin Gu [1,2], Huiyu Zhao [1,2], Peipei Qi [1,2], Zhiwei Wang [1,2], Zhenzhen Liu [1,2], Xinquan Wang [1,2,*,‡] and Shanshan Di [1,2,*,‡]

[1] State Key Laboratory for Quality and Safety of Agro-Products/Key Laboratory of Detection for Pesticide Residues and Control of Zhejiang, Institute of Agro-product Safety and Nutrition, Zhejiang Academy of Agricultural Sciences, Desheng Middle Road 298, Hangzhou 310021, China; chende@zaas.ac.cn (D.C.)

[2] Agricultural Ministry Key Laboratory for Pesticide Residue Detection, Hangzhou 310021, China

* Correspondence: wangxinquan212@163.com (X.W.); dishanshan1018@126.com (S.D.);
Tel.: +86-571-86419076 (X.W. & S.D.); Fax: +86-571-86419051 (X.W. & S.D.)

[†] These authors contributed equally to this work.

[‡] Study was a cooperative project. The two corresponding authors jointly administered, reviewed and editing the manuscript.

Number of Pages: 9

Number of Tables: 2

Number of Figures: 4

*2.1 Chemicals and Materials*

Sodium chloride (NaCl) and anhydrous magnesium sulfate ($MgSO_4$) were obtained from Huadong Medicine. Here, 2 mL tubes of purifying effect (Cleanert MAS-Q) composed of primary secondary amine (PSA, 50 mg), octadecylsilane (C18, 50 mg), and $MgSO_4$ (150 mg) were obtained from Tianjin Bonna-Agela Technologies Co., Ltd. (Tianjin, China). The carbon dioxide ($CO_2$) was obtained from Hangzhou Jingong Special Gas Co., Ltd. (China).

*2.3 Sample Preparation and Analysis*

Tetraconazole enantiomers analysis: The integrated QuEChERS method was used to prepare the rice and soil samples. First, 2.00 ± 0.01 g of samples, 2 mL of water, and 4 mL of acetonitrile were added. After vortexing for 1 min, 0.3 g of NaCl, 1.2 g of anhydrous $MgSO_4$, 10 mg of $Fe_3O_4$-PSA, 30 mg of C18, and 10 mg of GCB were added. Then, the tube was shaken vigorously and vortexed for 1 min. After placing the magnet outside the tube for 30 s, 0.6 mL of supernatant was transferred. The resulting solution was filtered (0.22 μm polytetrafluoroethylene filter prior) before SFC-MS/MS analysis.

The determination of tetraconazole enantiomers was carried out on SFC-MS/MS with an OZ-3 column (cellulose tris(3-chloro-4-methylphenyl carbamate), 3.0 × 150 mm, 3.0 μm, Daicel). As for the separation conditions, mobile phase, flow rate, column temperature, back pressure regulator (BPR) pressure, BPR temperature, and makeup solvent were $CO_2$/methanol (90:10, v/v), 3.0 mL/min, 40 °C, 10 MPa, 50 °C, and methanol containing 0.1% formic acid and 5 mM ammonium, respectively. In positive-ion mode, m/z 372.00 > 159.05 (–31 eV) and m/z 372.00 > 70.20 (–24 eV) were used for determination. The first peak was *R*-(-)-tetraconazole (0.93 min), and the second peak was *S*-(+)-tetraconazole (1.30 min). The analysis could be completed in 1.6 min. The recoveries were in the range of 86% to 112%.

Cd analysis: Cd concentrations in the plant samples were determined after digestion by $HNO_3$-$HClO_4$ (5:1, v:v) in a microwave digestion system. The concentration of Cd in soil was digestion by $HNO_3$-$HClO_4$-HF (5:1:1, v:v:v) in a microwave digestion system. The concentrations of Cd in the solutions were analyzed by ICP-MS (X-series 2, Thermo Fisher Scientific Inc., Massachusetts, USA). Reagent blanks and standard reference materials were added in each batch of digestion. The recoveries were in the range of 90% to 110%.

Soil property analysis: Soil pH was determined by a precision pH meter (PHS-3C, Rex, Shanghai) with a soil–water ratio of 1:2.5 (m: v). Soil organic matter content was measured using the modified Walkley–Black titrimetric procedure after potassium dichromate digestion. Total nitrogen was tested by the semi-micro Kjeldahl method, and total phosphorus was measured colorimetrically after $H_2SO_4$+$HClO_4$ digestion. Then, a 0.03 M $NH_4F$–0.025 M HCl solution was used to extract the available phosphorus and was determined colorimetrically.

*2.4. Determination of Soil Microorganisms*

The total genetic DNA of the samples was extracted and purified by the TIANamp Stool DNA Kit (Tiangen Biotech Co., Ltd, Beijing) and the AGENCOURTAMPURE XP Kit (Beckman Coulter, Inc., America), respectively. Amplification of the V3−V4 regions of the bacteria 16S rRNA gene was performed using primers B341F (CCTACGGGNGGCWGCAG) and B785R (GACTACHVGGGTATCTAATCC) as forward and reverse primers, respectively. Sequencing of the V3 and V4 regions was performed on an Illumina Miseq PE300 platform. The OUT's representative sequence had a confidence of greater than or equal to 0.97. PICRUSt was applied to obtain a functional profile from the 16S rRNA. Phylogenetic Investigation of Communities by Reconstruction of Unobserved States (PICRUSTs) and Kyoto Encyclopedia of Genes and Genomes (KEGG) databases were used to obtain functions for the genes that were predicted to be present in the samples and to assign the genes into metabolic pathways.

| | | | |
|---|---|---|---|
| KQ7d_4<br>ZY 18<br>Cd+$CaCO_3$ | KQ7d_3<br>ZG 96<br>Cd+$CaCO_3$ | **KQ7d_2**<br>**ZY 18**<br>**Cd** | **KQ7d_1**<br>**ZG 96**<br>**Cd** |
| KQ7d_8<br>ZY 18<br>Cd+$CaCO_3$+Tetrac<br>onazole | KQ7d_7<br>ZG 96<br>Cd+$CaCO_3$+Tetrac<br>onazole | KQ7d_6<br>ZY 18<br>Cd+Tetraconazole | KQ7d_5<br>ZG 96<br>Cd+Tetraconazole |

Fig. S1. The experimental design schematic diagram. The treatments of KQ7d_1, KQ7d_3, KQ7d_5, and KQ7d_7 were the exposure of Cd, Cd+$CaCO_3$, Cd+tetraconazole, and Cd+$CaCO_3$+tetraconazole on ZG 96, respectively. The treatments of KQ7d_2, KQ7d_4, KQ7d_6, and KQ7d_8 were the exposure of Cd, Cd+$CaCO_3$, Cd+tetraconazole, and Cd+$CaCO_3$+tetraconazole on ZY 18, respectively.

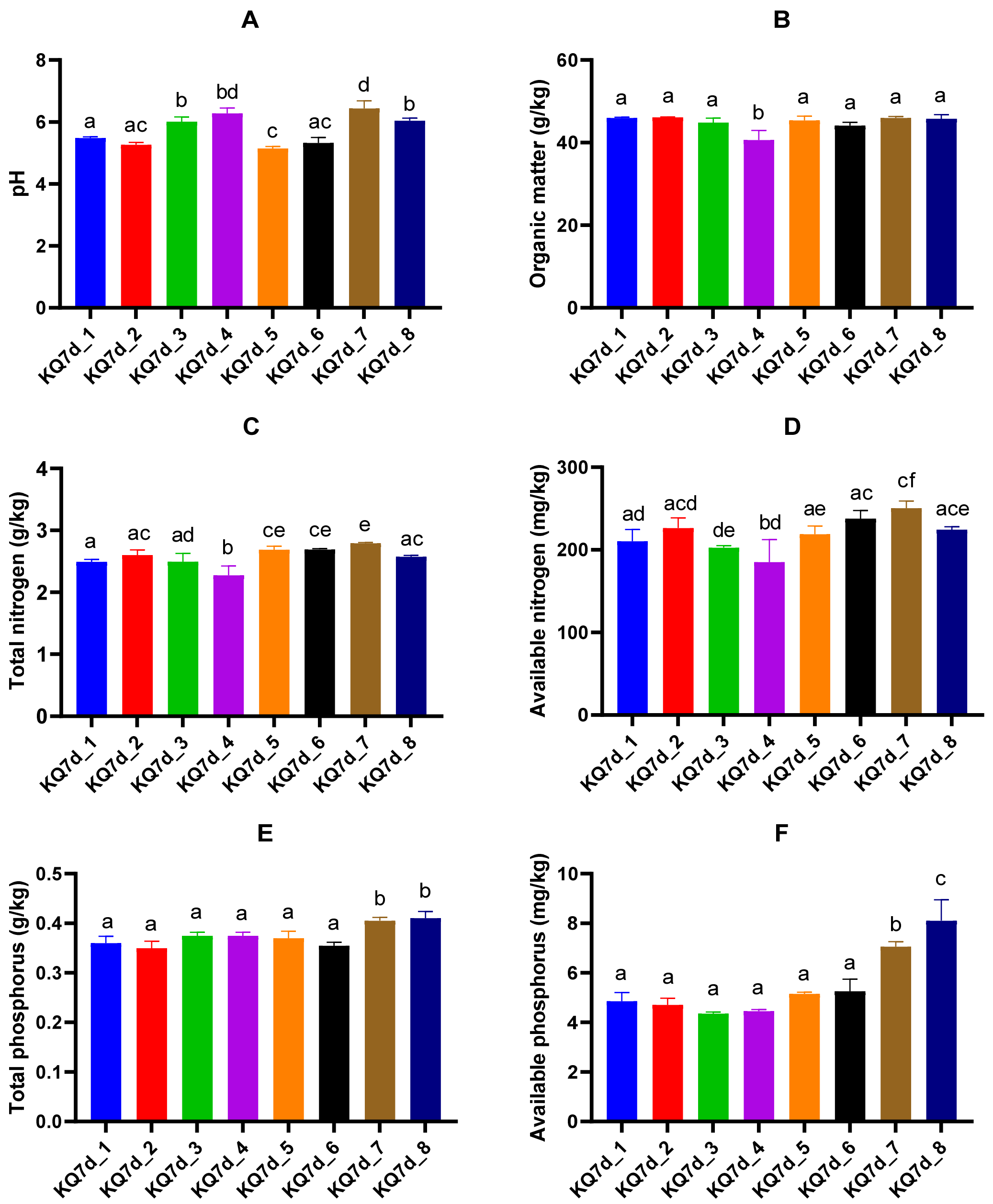


Fig. S2. The soil properties in different treatments. The different letters indicate the significant differences among different treatments ($p$ < 0.05). The treatments of KQ7d_1, KQ7d_3, KQ7d_5, and KQ7d_7 were the exposure of Cd, Cd+$CaCO_3$, Cd+tetraconazole, and Cd+$CaCO_3$+tetraconazole on ZG 96, respectively. The treatments of KQ7d_2, KQ7d_4, KQ7d_6, and KQ7d_8 were the exposure of Cd, Cd+$CaCO_3$, Cd+tetraconazole, and Cd+$CaCO_3$+tetraconazole on ZY 18, respectively.

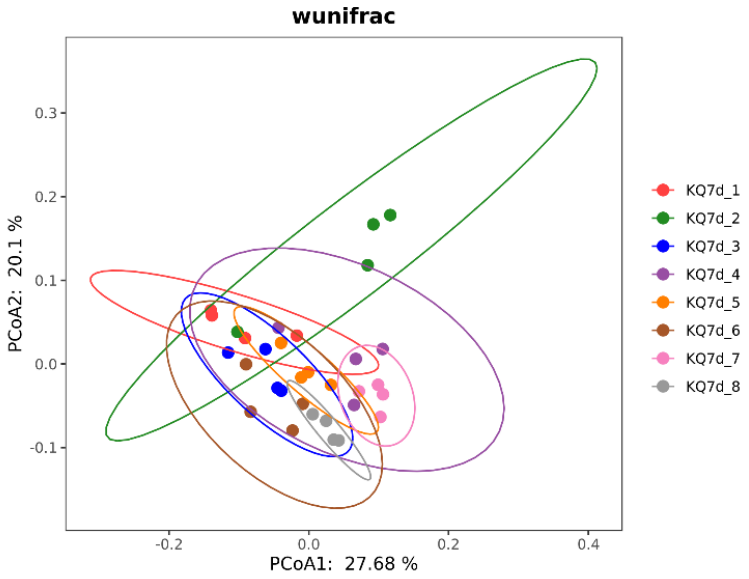


Fig. S3. Principal coordinate analysis (PCoA) of weighted UniFrac scores of soil microorganisms in different treatments. The treatments of KQ7d_1, KQ7d_3, KQ7d_5, and KQ7d_7 were the exposure of Cd, Cd+$CaCO_3$, Cd+tetraconazole, and Cd+$CaCO_3$+tetraconazole on ZG 96, respectively. The treatments of KQ7d_2, KQ7d_4, KQ7d_6, and KQ7d_8 were the exposure of Cd, Cd+$CaCO_3$, Cd+tetraconazole, and Cd+$CaCO_3$+tetraconazole on ZY 18, respectively.

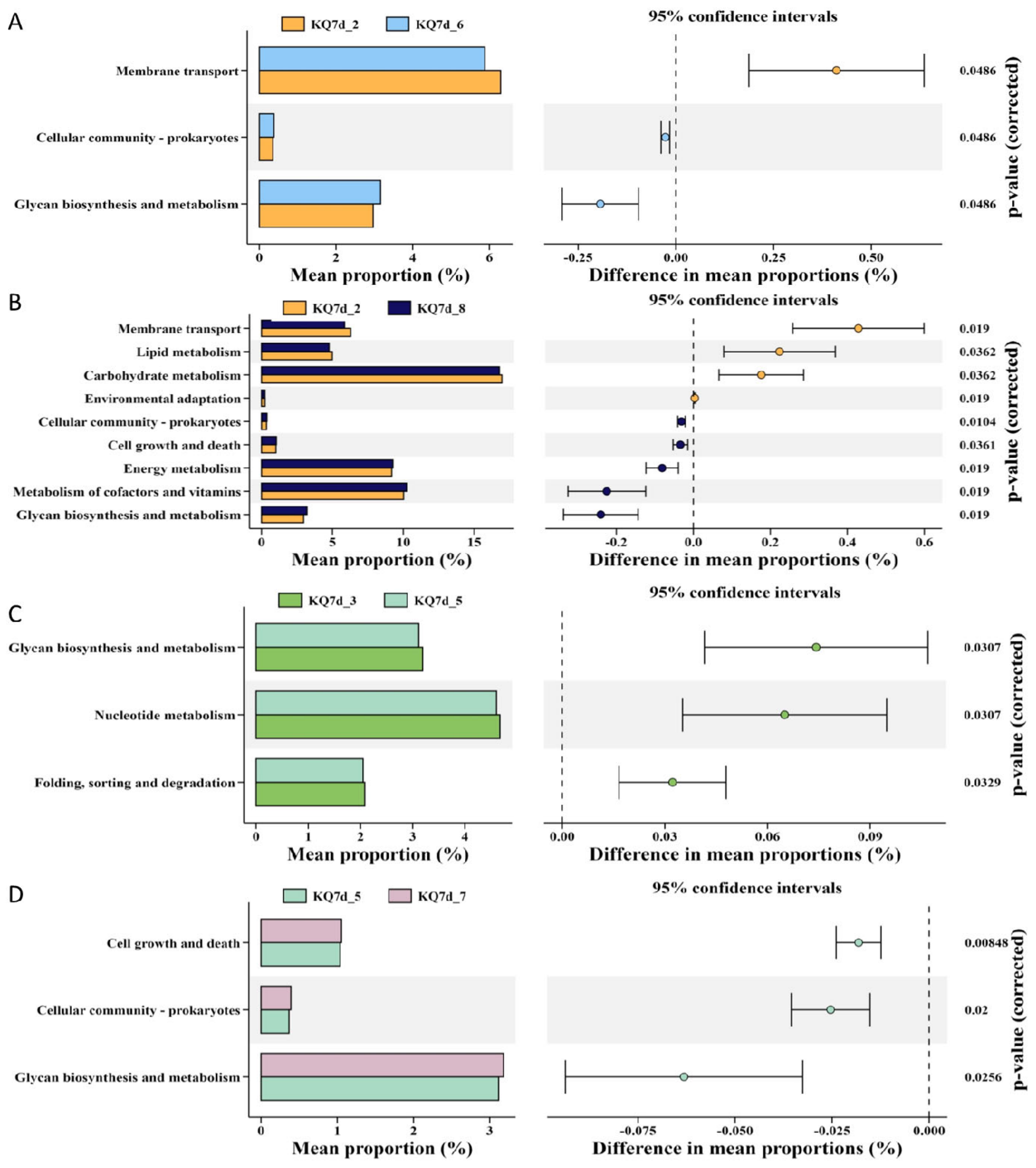


Fig. S4. The prediction analysis of KEGG pathways between different treatment groups at KEGG level 2: KQ7d_2 vs. KQ7d_6 (A), KQ7d_2 vs. KQ7d_8 (B), KQ7d_3 vs. KQ7d_5 (C), and KQ7d_5 vs. KQ7d_7 (D). The treatments of KQ7d_3, KQ7d_5, and KQ7d_7 were the exposure of Cd+$CaCO_3$, Cd+tetraconazole, and Cd+$CaCO_3$+tetraconazole on ZG 96, respectively. The treatments of KQ7d_2, KQ7d_6, and KQ7d_8 were the exposure of Cd, Cd+tetraconazole, and Cd+$CaCO_3$+tetraconazole on ZY 18, respectively.

Table S1. The relative abundance changes of microorganisms at the phylum level in different treatments. The treatments of KQ7d_1, KQ7d_3, KQ7d_5, and KQ7d_7 were the exposure of Cd, Cd+$CaCO_3$, Cd+tetraconazole, and Cd+$CaCO_3$+tetraconazole on ZG 96, respectively. The treatments of KQ7d_2, KQ7d_4, KQ7d_6, and KQ7d_8 were the exposure of Cd, Cd+$CaCO_3$, Cd+tetraconazole, and Cd+$CaCO_3$+tetraconazole on ZY 18, respectively.

| Phylum | KQ7d_1 | KQ7d_2 | KQ7d_3 | KQ7d_4 | KQ7d_5 | KQ7d_6 | KQ7d_7 | KQ7d_8 |
|---|---|---|---|---|---|---|---|---|
| *Bacteria_unclassified* | 0.3738 | 0.2786 | 0.3612 | 0.3454 | 0.3526 | 0.3477 | 0.3614 | 0.3789 |
| *Acidobacteria* | 0.2314 | 0.2149 | 0.2426 | 0.2090 | 0.2387 | 0.2592 | 0.2152 | 0.2491 |
| *Proteobacteria* | 0.2108 | 0.2677 | 0.2074 | 0.2475 | 0.2176 | 0.2190 | 0.2545 | 0.2213 |
| *Planctomycetes* | 0.0326 | 0.0436 | 0.0328 | 0.0295 | 0.0321 | 0.0259 | 0.0198 | 0.0173 |
| *Actinobacteria* | 0.0319 | 0.0556 | 0.0250 | 0.0272 | 0.0374 | 0.0257 | 0.0226 | 0.0234 |
| *Gemmatimonadetes* | 0.0308 | 0.0225 | 0.0420 | 0.0340 | 0.0284 | 0.0404 | 0.0238 | 0.0291 |
| *Chloroflexi* | 0.0242 | 0.0321 | 0.0216 | 0.0251 | 0.0270 | 0.0202 | 0.0270 | 0.0197 |
| *Verrucomicrobia* | 0.0211 | 0.0437 | 0.0202 | 0.0302 | 0.0280 | 0.0155 | 0.0240 | 0.0207 |

Table S2. The relative abundance changes of microorganisms at the genus level in different treatments. The treatments of KQ7d_1, KQ7d_3, KQ7d_5, and KQ7d_7 were the exposure of Cd, Cd+$CaCO_3$, Cd+tetraconazole, and Cd+$CaCO_3$+tetraconazole on ZG 96, respectively. The treatments of KQ7d_2, KQ7d_4, KQ7d_6, and KQ7d_8 were the exposure of Cd, Cd+$CaCO_3$, Cd+tetraconazole, and Cd+$CaCO_3$+tetraconazole on ZY 18, respectively.

| Genus | KQ7d_1 | KQ7d_2 | KQ7d_3 | KQ7d_4 | KQ7d_5 | KQ7d_6 | KQ7d_7 | KQ7d_8 |
|---|---|---|---|---|---|---|---|---|
| *Bacteria_unclassified* | 0.3738 | 0.2786 | 0.3612 | 0.3454 | 0.3526 | 0.3477 | 0.3614 | 0.3789 |
| *Deltaproteobacteria_unclassified* | 0.0518 | 0.0387 | 0.0546 | 0.0395 | 0.0451 | 0.0555 | 0.0413 | 0.0443 |
| *Gp7_unclassified* | 0.0424 | 0.0282 | 0.0416 | 0.0281 | 0.0330 | 0.0330 | 0.0233 | 0.0283 |
| *Gp16_unclassified* | 0.0391 | 0.0376 | 0.0425 | 0.0225 | 0.0321 | 0.0347 | 0.0215 | 0.0228 |
| *Gp1_unclassified* | 0.0335 | 0.0391 | 0.0388 | 0.0445 | 0.0511 | 0.0622 | 0.0420 | 0.0561 |
| *Betaproteobacteria_unclassified* | 0.0288 | 0.0494 | 0.0264 | 0.0421 | 0.0324 | 0.0307 | 0.0461 | 0.0342 |
| *Gp18_unclassified* | 0.0259 | 0.0182 | 0.0314 | 0.0254 | 0.0251 | 0.0333 | 0.0303 | 0.0323 |
| *Gp3_unclassified* | 0.0236 | 0.0222 | 0.0228 | 0.0271 | 0.0280 | 0.0315 | 0.0311 | 0.0355 |
| *Myxococcales_unclassified* | 0.0215 | 0.0224 | 0.0161 | 0.0246 | 0.0172 | 0.0179 | 0.0211 | 0.0183 |
| *Gemmatimonadetes_unclassified* | 0.0208 | 0.0136 | 0.0247 | 0.0182 | 0.0191 | 0.0224 | 0.0135 | 0.0187 |
| *Gp6_unclassified* | 0.0203 | 0.0234 | 0.0219 | 0.0147 | 0.0206 | 0.0172 | 0.0190 | 0.0226 |
| *Isosphaeraceae_unclassified* | 0.0202 | 0.0267 | 0.0180 | 0.0137 | 0.0197 | 0.0143 | 0.0090 | 0.0073 |
| *Rhodospirillales_unclassified* | 0.0144 | 0.0207 | 0.0177 | 0.0193 | 0.0191 | 0.0214 | 0.0190 | 0.0186 |
| *Subdivision3_unclassified* | 0.0143 | 0.0331 | 0.0147 | 0.0223 | 0.0195 | 0.0104 | 0.0183 | 0.0157 |
| *Proteobacteria_unclassified* | 0.0139 | 0.0121 | 0.0155 | 0.0139 | 0.0158 | 0.0139 | 0.0198 | 0.0161 |
| *Actinobacteria_unclassified* | 0.0117 | 0.0198 | 0.0070 | 0.0075 | 0.0123 | 0.0086 | 0.0078 | 0.0077 |
| *Rhizobiales_unclassified* | 0.0109 | 0.0153 | 0.0101 | 0.0072 | 0.0109 | 0.0083 | 0.0078 | 0.0086 |
| *Chloroflexi_unclassified* | 0.0100 | 0.0124 | 0.0087 | 0.0072 | 0.0088 | 0.0077 | 0.0089 | 0.0075 |
